# The Progression of Xylem Vessel Cell Differentiation is Dependent on the Activity Level of VND7 in *Arabidopsis thaliana*

**DOI:** 10.3390/plants9010039

**Published:** 2019-12-25

**Authors:** Risaku Hirai, Takumi Higaki, Yuto Takenaka, Yuki Sakamoto, Junko Hasegawa, Sachihiro Matsunaga, Taku Demura, Misato Ohtani

**Affiliations:** 1 Division of Biological Science, Graduate School of Science and Technology, Nara Institute of Science and Technology, Ikoma 630-0192, Japan; hirai.risaku.hk1@bs.naist.jp (R.H.); yuto-t@fc.ritsumei.ac.jp (Y.T.); 2 International Research Organization for Advanced Science and Technology, Kumamoto University, Kumamoto 860-8555, Japan; thigaki@kumamoto-u.ac.jp; 3 Faculty of Science and Technology, Department of Applied Biological Science, Tokyo University of Science, Noda 278-8510, Japan; yuki_sakamoto@bio.sci.osaka-u.ac.jp (Y.S.); 6414702@almuni.tus.ac.jp (J.H.); sachi@rs.tus.ac.jp (S.M.); 4 Department of Integrated Biosciences, Graduate School of Frontier Sciences, The University of Tokyo, Kashiwa 277-8562, Japan

**Keywords:** VASCULAR-RELATED NAC-DOMAIN7, xylem vessel cell, programmed cell death, secondary cell wall, transcriptional regulation, *Arabidopsis thaliana*

## Abstract

Xylem vessels are important for water conduction in vascular plants. The VASCULAR-RELATED NAC-DOMAIN (VND) family proteins, master regulators of xylem vessel cell differentiation in *Arabidopsis thaliana*, can upregulate a set of genes required for xylem vessel cell differentiation, including those involved in secondary cell wall (SCW) formation and programmed cell death (PCD); however, it is not fully understood how VND activity levels influence these processes. Here, we examined the *Arabidopsis VND7-VP16-GR* line, in which VND7 activity is post-translationally activated by treatments with different concentrations of dexamethasone (DEX), a synthetic glucocorticoid. Our observations showed that 1 nM DEX induced weak SCW deposition, but not PCD, whereas 10 or 100 nM DEX induced both SCW deposition and PCD. The decreased chlorophyll contents and SCW deposition were apparent after 24 h of 100 nM DEX treatment, but became evident only after 48 h of 10 nM DEX treatment. Moreover, the lower DEX concentrations delayed the upregulation of VND7 downstream genes, and decreased their induction levels. They collectively suggest that the regulation of VND activity is important not only to initiate xylem vessel cell differentiation, but also regulate the quality of the xylem vessels through VND-activity-dependent upregulation of the PCD- and SCW-related genes.

## 1. Introduction

Water conduction is crucial for terrestrial plant species living on dry land. To increase water conductive activity, vascular plants developed specialized conductive tissues called xylem vessels, which are characterized by secondary cell wall (SCW) deposition and programmed cell death (PCD) [1,2]. Because of the importance of xylem vessels, great efforts have been made to understand the molecular mechanisms underlying their formation, revealing, for example, that phytohormonal regulation involving auxin, cytokinins, and brassinosteroids is critical for inducing the differentiation of xylem vessel cells [3,4,5,6,7,8].

One of the remarkable achievements of molecular biological research into xylem vessel formation in *Arabidopsis thaliana* was the identification of the master transcriptional regulators of their differentiation, the VASCULAR-RELATED NAC-DOMAIN (VND) family proteins [9]. The *Arabidopsis* VND family contains seven members (VND1 to VND7), and overexpression analyses revealed that all *VND* genes possess the ability to induce xylem vessel cell differentiation [9,10,11,12,13]. A detailed analysis of the pathway functioning downstream of the VND family proteins demonstrated that they directly and/or indirectly upregulate the entire sets of genes required for SCW deposition and PCD [6,14,15,16,17]. The upregulation of the SCW-related genes by the VND proteins is partially mediated by the secondary master regulators MYB46 and MYB83 [18,19,20,21], and multiple MYB transcription factors also function downstream in the SCW biosynthesis pathway [14,22,23,24]. Functional analyses of the *VND* and *MYB* gene homologs suggested that the VND-MYB-based transcriptional network is well conserved among land plants [14,15], including moss (*Physcomitrella patens*) [25], pine (*Pinus taeda*) [26], poplar (*Populus trichocarpa*) [27,28], and rice (*Oryza sativa*) [29].

In addition, several other proteins have been reported to transcriptionally or post-transcriptionally regulate the function of the VND family proteins. The LOB-DOMAIN proteins LBD30, LBD18, and LBD15 form a positive transcriptional feedback loop with VND7: the expression of the *LBD* genes is regulated by VND7, but they, in turn, can upregulate *VND7* expression [10,30,31]. VND-INTERACTING 2 (VNI2), a distinct NAC-domain protein, binds directly to VND7 to suppress its transactivation activity [32]. Moreover, a forward genetics approach revealed the importance of the post-translational regulation of VND7 activity to reflect cellular redox condition via the *S-*nitrosylation of specific cysteine residues in its C-terminal region [33,34]. In addition, light modulates VND1–3 activity to regulate the venation of cotyledons during seedling development [7]. These findings collectively suggest that xylem vessel cell differentiation is controlled by multiple layers of VND-related regulation, optimizing xylem vessel function in response to the environmental conditions [15]. Despite these discoveries, it is not yet clear how the levels of VND activity influence this transcriptional network and promote xylem vessel cell differentiation.

**Figure 1:**
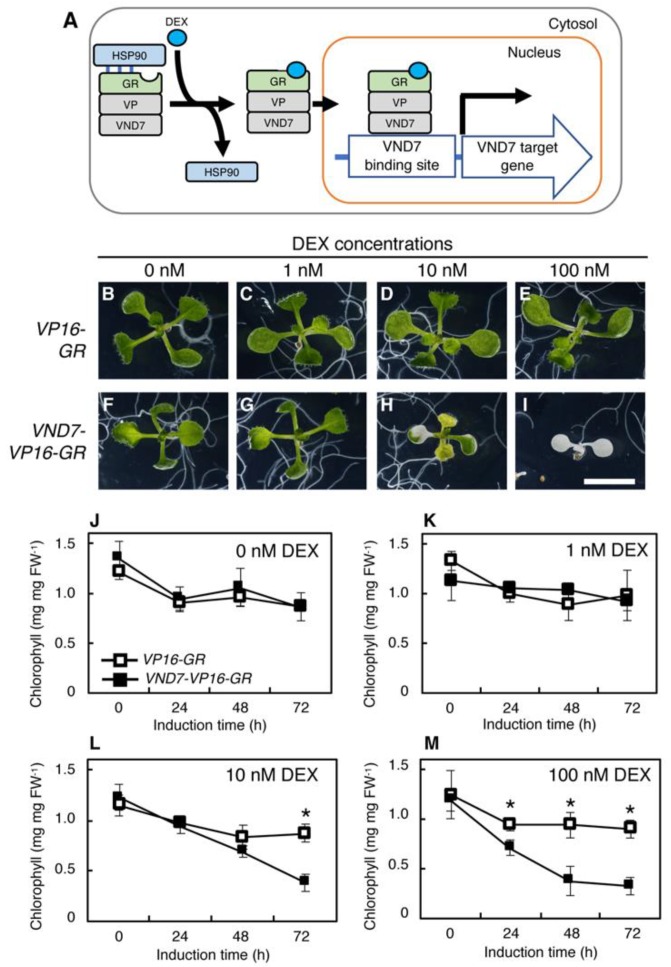
The concentration of dexamethasone (DEX) affects the progression of programmed cell death in *VND7-VP16-GR*. (**A**) The schematic molecular mode of action of the *VND7-VP16-GR* system. The chimeric VND7-VP16-GR protein is localized in the cytosol in the absence of a glucocorticoid, since the glucocorticoid receptor (GR) interacts with heat shock protein 90 (HSP90) to prevent its entry into the nucleus. In the presence of glucocorticoid molecules, such as the synthetic glucocorticoid DEX, GR changes its structure to release HSP90, allowing the VND7-VP16-GR protein to localize to the nucleus. This resulted in the activation of the transcriptional activity of VND7 [12]. (**B**–**I**) Effects of different DEX concentrations on the growth and morphology of *VP16-GR* (vector control) and *VND7-VP16-GR* seedlings. The 7-day-old *VP16-GR* (B–E) and *VND7-VP16-GR* (**F**–**I**) seedlings were treated with 0, 1, 10, and 100 nM DEX for 3 d. Bar = 5 mm. (**J**–**M**) Changes in the chlorophyll amounts of *VP16-GR* and *VND7-VP16-GR* seedlings. The 7-day-old *VP16-GR* and *VND7-VP16-GR* seedlings were treated with 0, 1, 10, and 100 nM DEX, and sampled after 0, 24, 48, and 72 h. The data are shown as means ± SD (*n* = 4). Asterisks indicate statistically significant differences between *VP16-GR* and *VND7-VP16-GR* (Student’s *t*-test, *p* < 0.05).

To elucidate the role of VND activity levels in xylem vessel differentiation, we utilized the *VND7-VP16-GR* system, an inducible VND7-activation line in which ectopic xylem vessel cell differentiation is induced by treatment with a glucocorticoid [12]. In this system, part of the rat glucocorticoid receptor (GR) protein is fused with VND7 and VP16, a strong transcriptional activator domain. The GR interacts with heat shock protein 90 (HSP90) to prevent its entry into the nucleus, but once a glucocorticoid molecule, such as the synthetic glucocorticoid dexamethasone (DEX), binds to the GR, the VND7-VP16-GR protein moves from the cytosol into the nucleus to function as a transcription factor (Figure 1A) [12]. Previous studies showed glucocorticoid dose-dependent activation of GR-fused chimeric transcription factor proteins in plant cells [35,36], suggesting that the *VND7-VP16-GR* system would be useful for testing the responses of plant cells to different levels of VND7 activity. Here, we monitored the decreases in chlorophyll contents (an indicator of PCD) and the efficiency of SCW deposition after treatment of *VND7-VP16-GR-*expressing transgenic plants with different concentrations of DEX. Our data showed that the different DEX concentrations differentially induced xylem vessel differentiation through changes in the expression patterns of genes downstream of VND7, suggesting that the regulation of VND7 activity level is critical in xylem cell formation.

## 2. Results and Discussion

### 2.1. Lower DEX Concentrations Alter the Progression of PCD in VND7-VP16-GR

To elucidate the effects of differences in VND7 activity on the differentiation of the xylem vessel cells, we treated the *VND7-VP16-GR* seedlings with different concentrations of DEX (Figure 1), using the *VP16-GR* seedlings as the vector control. Previously, *VND7-VP16-GR* plants were treated with 10 µM DEX, which effectively induced the differentiation of the xylem vessel cells [12,33]. Here, we used DEX concentrations of 100, 10, 1, and 0 nM (Figure 1B–I). In the vector control plants *VP16-GR*, no visible difference was detected by any conditions of DEX treatment (Figure 1B–E). We observed that 100 nM DEX induced PCD in the 7-day-old *VND7-VP16-GR* seedlings, causing them to become completely bleached and die (Figure 1I). By contrast, 10 nM DEX and 1 nM DEX only partially (Figure 1H) and rarely (Figure 1G) induced this bleaching of the leaves, respectively. Seedling growth was also differently affected by the DEX concentration; 1 nM DEX did not affect growth (Figure 1G), but the seedlings treated with 10 nM DEX were smaller than the seedlings treated with 0 nM DEX (Figure 1H). Thus, 1 nM DEX did not induce PCD in the 7-day-old *VND7-VP16-GR* seedlings.

We further checked the amount of chlorophyll in the seedling extracts to quantitatively explore the effects of the DEX concentration (Figure 1J–M). In accordance with the observations described above, the chlorophyll contents of the *VND7-VP16-GR* seedlings treated with 0 and 1 nM DEX were comparable to the vector controls at all time points (Figure 1J,K). By contrast, a decreased chlorophyll content was evident after 72 h of the 10 DEX treatment or 24 h of the 100 nM DEX treatment (Figure 1L,M), although both of these treatments resulted in similar chlorophyll contents after 72 h. These results suggest that the lower the DEX concentration, the slower the progression of PCD.

### 2.2. Lower DEX Concentrations Affect the Progression of SCW Deposition

**Figure 2:**
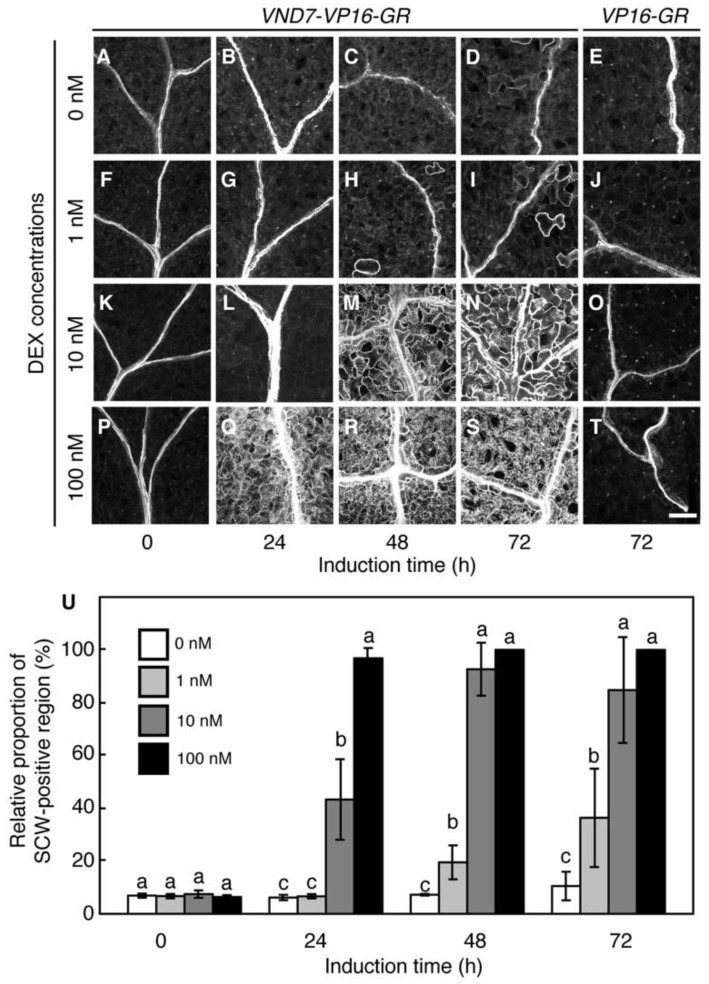
The concentration of dexamethasone (DEX) affects the progression of secondary cell wall (SCW) deposition in *VND7-VP16-GR*. (**A**–**T**) Seven-day-old *VP16-GR* (**E**,**J**,**O**,**T**) and *VND7-VP16-GR* (**A**–**D**,**F**–**I**,**K**–**N**,**P**–**S**) seedlings were treated with 0, 1, 10, and 100 nM DEX, and sampled after 0, 24, 48, and 72 h. The cotyledons were fixed and stained with propidium iodide to visualize the SCW, then embedded in the TOMEI solution [37]. The samples were observed using a confocal microscope. In the case of *VP16-GR*, only samples incubated for 72 h were shown. Bar = 100 µm. (**U**) Changes in the relative proportion of SCW-positive cells within the cotyledons. The relative proportions of SCW-positive cell regions were calculated manually using an image analysis performed in ImageJ. The data are shown as means ± SD (*n* = 5). Different letters indicate statistically significant values (Tukey–Kramer test, *p* < 0.05).

Next, we examined the DEX-concentration dependency of ectopic SCW deposition in the *VND7-VP16-GR* cotyledons. The TOMEI-I method [37] was utilized for the visualization of SCW deposition (Figure 2A–T). No clear deposition of ectopic SCW was observed in the *VND7-VP16-GR* plants treated with the mock control (Figure 2A–E); however, following 48 h treatment with 1 nM DEX, a few SCW-positive cells could be detected (Figure 2F–J). By contrast, the 10 and 100 nM DEX treatments induced the deposition of thick and patterned SCW after 48 h and 24 h of treatment, respectively (Figure 2K–T; Supplementary Figure S1). The relative proportion of the SCW-positive region was calculated manually (Figure 2U) or semi-automatically based on machine learning (Supplementary Figures S2 and S3) [38]. Both methods demonstrated that the 1, 10, and 100 nM DEX treatments could induce the ectopic deposition of SCW after 72 h of treatment, although the thickness of the deposited SCW, as reflected by signal intensity, was lower in the samples treated with 1 nM DEX than those treated with 10 or 100 nM DEX (Figure 2I,N; Supplementary Figure S2). The time-course analysis showed that the relative proportion of SCW positive region was nearly reached its peak after 24 h of the 100 nM DEX treatment (Figure 2Q,U), while the SCW-positive region was less than 50% of leaf area after a 24 h treatment with 10 nM DEX and peaked at 48 h (Figure 2L,M,U). These observations clearly indicate that the DEX concentration affects not only the progression of PCD, but also that of SCW deposition.

The seedling growth and chlorophyll content suggested that 1 nM DEX did not induce PCD in *VND7-VP16-GR* (Figure 1G,K); however, this treatment caused the ectopic deposition of a thin layer of SCW in the *VND7-VP16-GR* cotyledons, although not as much as was induced by the 10 nM or 100 nM DEX treatments (Figure 2I,U). These findings could reflect a difference in the VND7 activity-dependency between PCD and SCW deposition. The genes encoding the PCD-related enzymes are directly activated by the VND proteins, whereas the SCW-related genes are under the control of not only the VND proteins [6,16,17], but also MYB46 and MYB83 [20,39]. It is therefore possible that the different effects of the 1 nM DEX treatment could be caused by the different transcriptional regulatory networks involved in PCD and SCW deposition.

### 2.3. Different DEX Treatments Altered the Expression Patterns of the Genes Downstream of VND7

**Figure 3:**
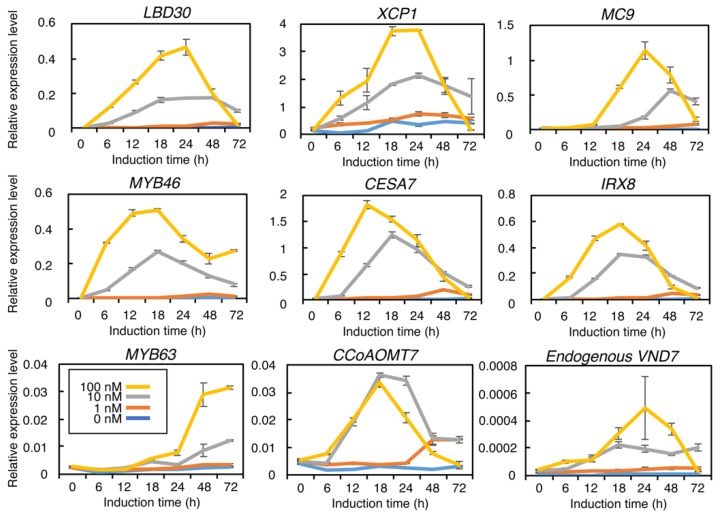
Quantitative RT-PCR analysis of the genes downstream of VND7 in *VND7-VP16-GR* treated with different concentrations of dexamethasone (DEX). Seven-day-old *VND7-VP16-GR* seedlings were treated with 0, 1, 10, and 100 nM DEX, and sampled after 0, 6, 12, 18, 24, 48, and 72 h. The expression levels of the genes downstream of VND7 were normalized to the expression level of the internal control *UBIQUITIN10*. The data are shown as means ± SD (*n* = 3).

To elucidate the VND7-activity-dependent activation of the genes downstream of this protein, we performed a quantitative RT-PCR analysis on selected genes known to function in this pathway (Figure 3; Supplementary Figure S4) [16,24,33,40]. We selected the PCD-related enzyme genes *XYLEM CYSTEINE PEPTIDASE1* (*XCP1*) and *METACASPASE9* (*MC9*) [40,41], the SCW-related transcription factor-encoding genes *MYB46* and *MYB63* [18,19,20,24,42], and the SCW biosynthetic genes, including *CELLULOSE SYNTHASE7/IRREGULAR XYLEM3* (*CESA7/IRX3*) for cellulose biosynthesis, *IRREGULAR XYLEM8* (*IRX8*) for xylan biosynthesis, and *CAFFEOYL COENZYME A ESTER O-METHYLTRANSFERASE*7 (*CCoAOMT7*) for lignin biosynthesis [43,44,45,46]. In addition, we determined the expression of *LBD30* [31] and the endogenous *VND7* gene [9]. 

Our time-course quantitative RT-PCR analysis revealed clear differences in the expression patterns of the genes downstream of VND7 following the different DEX treatments (Figure 3). The 10 and 100 nM DEX treatments led to a greater than ten-fold upregulation of these genes; however, 1 nM DEX did not strongly increase the gene expression levels (Figure 3; Supplementary Figure S4). Moreover, except for *CCoAOMT7*, the expression peaks of the genes downstream of VND7 were higher and reached more quickly in the plants treated with 100 nM DEX than in the plants treated with 10 nM DEX (Figure 3). These observations were consistent with the chlorophyll contents and SCW depositions observed following these treatments (Figure 1 and Figure 2); therefore, the progression of PCD and SCW is likely directly linked to the VND7-regulated activation of these downstream genes.

**Figure 4:**
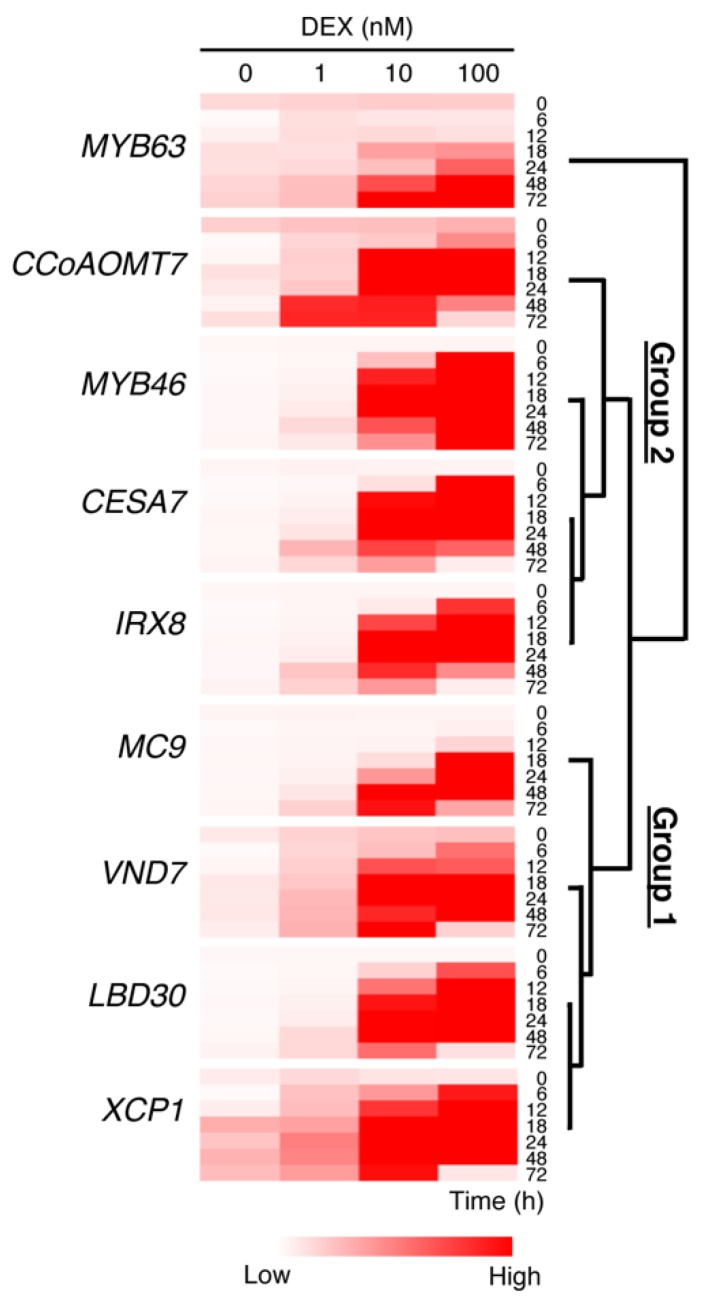
Hierarchical clustering analysis of the expression patterns of the genes downstream of VND7. The hierarchical clustering analysis was performed using the normalized quantitative RT-PCR data (Figure 3).

Previous studies revealed that *LBD30*, *MYB46*, *XCP1*, and *MC9* are direct targets of VND7 [6,16,17], while *MYB63*, *CESA7*, and *IRX8* are direct targets of MYB46 [18,21]. In addition, the promoter region of *CCoAOMT7* contains the sequence ACCAACC, which can be recognized by both MYB46 and MYB83 [18,21,42], suggesting that this gene might be directly regulated by these transcription factors. To test the effects of the DEX treatments on these transcriptional regulatory relationships, we performed a hierarchical clustering analysis on the gene expression patterns (Figure 4). The results clearly separated the tested genes into three groups based on their similarities in expression pattern: group 1 contained *VND7*, *LBD30*, *XCP1*, and *MC9*; group 2 comprised *MYB46*, *CESA7*, *IRX8*, and *CCoAOMT7*; and group 3 contained *MYB63* alone (Figure 4), reflecting the transcriptional hierarchy of VND7, MYB46, and MYB63 [14,15,19,47]. The level of VND7 activity can therefore affect the induction of gene expression in a transcriptional regulatory hierarchical manner.

We also observed that the *MYB63* gene, encoding one of the third-layer transcription factors in the VND-MYB-based transcriptional network regulating lignin biosynthesis [24], showed an expression pattern distinct from that of the other genes at the transcriptional level (Figure 3 and Figure 4). *MYB63* expression was induced after 24 h and 48 h of the 100 nM and 10 nM DEX treatments, respectively, and continuously increased during 72 h of these treatments, whereas the other genes were transiently upregulated by these treatments (Figure 3 and Figure 4). These observations support the hypothesis that the step-wise induction of the MYB transcription factors can be effective for regulating lignin biosynthesis temporally and flexibly to adapt the plant to its environmental conditions [15,23].

## 3. Materials and Methods

### 3.1. Plant Materials and Growth Conditions

The transgenic *Arabidopsis thaliana* lines (Col-0 background) expressing either *VND7-VP16-GR* or *VP16-GR* were those described by [12]. The *VND7-VP16-GR* and *VP16-GR* seedlings were grown under continuous light at 22 °C for 7 d on Murashige and Skoog medium (Wako, Osaka, Japan) containing 1% (*w/v*) sucrose (nacalai tesque, Kyoto, Japan), 0.05 (*w/v*) MES (nacalai tesque), and 0.6% (*w/v*) Gellan gum (Wako), adjusted to pH 5.7.

### 3.2. DEX Treatment

The 7-day-old seedlings were treated with 0, 1, 10, or 100 nM DEX (Sigma-Aldrich, St. Louis, MO, USA), as previously described [33,48]. Briefly, the DEX solution was poured directly onto the seedling plate to cover the seedlings, after which the plates were incubated under continuous light at 22 °C. The seedlings were then sampled at the indicated time points.

### 3.3. Chlorophyll Content Measurement

Chlorophyll was extracted from the DEX-treated seedlings using an overnight incubation in *N*,*N*-dimethylformamide at 4 °C. The chlorophyll contents were calculated as described by [49], using absorbance data obtained from a spectrometry analysis using a DU640 spectrophotometer (Beckman Coulter, Brea, CA, USA).

### 3.4. Microscope Observation

The DEX-treated seedlings were sampled and fixed in a mixture of 90% (*w/v*) ethanol and 10% (*w/v*) acetic acid. The samples were then washed three times with 90% (*w/v*) ethanol and stained with a 5 µg mL^−1^ propidium iodide (PI) solution. The PI-stained samples were cleared using TOMEI-I, as described by [37], and were then observed using a confocal microscope (FV-10i; Olympus, Tokyo, Japan). 

### 3.5. Calculation of the Relative Proportion of SCW-Positive Cell Regions in the Cotyledons

To calculate the relative proportion of SCW deposition in cotyledons, the area displaying a PI signal was manually measured, or semi-automatically measured based on machine learning [38]. For the manual measurements, the confocal images were processed using ImageJ (https://imagej.nih.gov/ij/index.html) to obtain a maximum intensity projection image. The obtained images of partial cotyledon regions were then merged to generate an image of the whole cotyledon using the MosaicJ plugin [50]. The area of the merged image displaying the SCW signals was measured, and the relative proportion of the SCW-positive region as a proportion of the total area of each cotyledon was calculated.

For the semi-automatic detection of SCW deposition, the previously reported image analysis framework for object detection in transmission electron microscopic images was used [38]. First, square regions of interest (ROIs) (50 × 50 pixels) were tiled on a maximum intensity projection image of the PI-stained cotyledon (3040 × 3888 pixels) (Figure S2A, top). Next, the ROIs with a maximum intensity of 254 or 255 were selected as candidates for SCW-positive regions (Figure S2A, bottom). Cluster analyses of the candidate ROIs were performed using a self-organizing map (node number: 15 × 15) with statistical geometric features [51]. The feature set was optimized to detect the SCW based on interactive annotations and iterative clustering with a genetic algorithm (Figure S2B) [38]. After the feature selection, the SCW-positive regions were automatically detected in the original PI images. The endogenous SCW-positive regions were eliminated using the mathematical morphological operators of erosion and dilation (Figure S3A).

### 3.6. Quantitative RT-PCR Analysis

The quantitative RT-PCR analysis was performed as described by [16]. Briefly, the seedlings were collected at the indicated time after the DEX treatment, then ground in liquid nitrogen using a TissueLyser II (Qiagen, Hilden, Germany). Total RNA was isolated using an RNeasy Plant Mini Kit (Qiagen), according to the manufacturer’s instructions. The cDNA synthesis was performed with 1 µg total RNA using a Transcriptor First-Strand cDNA Synthesis kit (Roche, Basel, Switzerland). The quantitative PCR was carried out using a LightCycler 480 DNA SYBR Green system (Roche). The expression levels of the genes of interest were normalized to the expression level of the internal control, *UBIQUITIN10* (*UBQ10*). The primers used in this study were shown in Table S1 [16,24,33,40].

### 3.7. Hierarchical Clustering Analysis of the Expression of Genes Downstream of VND7

The quantitative RT-PCR data were normalized, then used for a hierarchical clustering analysis (Pearson correlation; average linkage clustering method) performed using the software MeV ver. 4.9.0 [52]. The heatmap visualization of the quantitative RT-PCR data was also created using MeV ver. 4.9.0.

## 4. Conclusions

Our data suggest that the level of VND activity can affect the progression of xylem vessel cell differentiation. The different levels of VND activity can differentially induce the PCD- and SCW-related genes (Figure 3 and Figure 4), resulting in differences in the progression of PCD and SCW deposition (Figure 1 and Figure 2). These findings also suggest that the regulation of VND activity is important not only to control the initiation of the molecular programs of xylem vessel cell differentiation, but also regulate the functional quality of the xylem vessels to change how PCD and SCW deposition progress. Previous works have revealed several post-translational regulatory mechanisms affecting VND7 activity, such as the physical interaction with the repressor protein VNI2 [32] and the *S-*nitrosylation of the VND7 protein [33,34]; however, the molecular evidence to link these regulatory mechanisms to xylem vessel quality is still not well studied. Further studies on the regulatory mechanisms of VND activity could provide new opportunities for biotechnologies to increase crop yields and plant biomass by influencing xylem vessel quality.

## Supplementary Materials

The following are available online at https://www.mdpi.com/2223-7747/9/1/39/s1, Table S1. Primers used in this study; Figure S1. Secondary cell wall (SCW) deposition in *VND7-VP16-GR* following a 24 h treatment with 10 nM dexamethasone (DEX); Figure S2. Image processing workflow for the semi-automatic detection of the secondary cell wall (SCW)-positive regions; Figure S3. The relative proportion of secondary cell wall (SCW)-positive cells within *VP16-GR* and *VND7-VP16-GR* seedlings; Figure S4. Quantitative RT-PCR analysis of the expression of the genes downstream of VND7 in *VND7-VP16-GR* seedlings treated with 0 and 1 nM dexamethasone (DEX).

## References

[1] Sperry J.S. (2003). Evolution of water transport and xylem structure. Int. J. Plant Sci..

[2] Turner S., Gallois P., Brown D. (2007). Tracheary element differentiation. Annu. Rev. Plant Biol..

[3] Fukuda H., Komamine A. (1980). Establishment of an experimental system for the study of tracheary element differentiation from single cells isolated from the mesophyll of *Zinnia elegans*. Plant Physiol..

[4] Koizumi K., Sugiyama M., Fukuda H. (2000). A series of novel mutants of *Arabidopsis thaliana* that are defective in the formation of continuous vascular network: Calling the auxin signal flow canalization hypothesis into question. Development.

[5] Kondo Y., Nurani A.M., Saito C., Ichihashi Y., Saito M., Yamazaki K., Mitsuda N., Ohme-Takagi M., Fukuda H. (2016). Vascular cell induction culture system using Arabidopsis leaves (VISUAL) reveals the sequential differentiation of sieve element-like cells. Plant Cell.

[6] Ohashi-Ito K., Oda Y., Fukuda H. (2010). *Arabidopsis* VASCULAR-RELATED NAC-DOMAIN6 directly regulates the genes that govern programmed cell death and secondary wall formation during xylem differentiation. Plant Cell.

[7] Tan T.T., Endo H., Sano R., Kurata T., Yamaguchi M., Ohtani M., Demura T. (2018). Arabidopsis VND1–VND3 contribute to xylem vessel element formation in the cotyledon. Plant Physiol..

[8] Yamamoto R., Demura T., Fukuda H. (1997). Brassinosteroids induce entry into the final stage of tracheary element differentiation in cultured *Zinnia* cells. Plant Cell Physiol..

[9] Kubo M., Udagawa M., Nishikubo N., Horiguchi G., Yamaguchi M., Ito J., Mimura T., Fukuda H., Demura T. (2005). Transcription switches for protoxylem and metaxylem vessel formation. Genes Dev..

[10] Endo H., Yamaguchi M., Tamura T., Nakano Y., Nishikubo N., Yoneda A., Kato K., Kubo M., Kajita S., Katayama Y. (2015). Multiple classes of transcription factors regulate the expression of VASCULAR-RELATED NAC-DOMAIN7, a master switch of xylem vessel differentiation. Plant Cell Physiol..

[11] Yamaguchi M., Kubo M., Fukuda H., Demura T. (2008). VASCULAR-RELATED NAC-DOMAIN7 is involved in the differentiation of all types of xylem vessels in Arabidopsis roots and shoots. Plant J..

[12] Yamaguchi M., Goué N., Igarashi H., Ohtani M., Nakano Y., Mortimer J.C., Nishikubo N., Kubo M., Katayama Y., Kakegawa K. (2010). VASCULAR-RELATED NAC-DOMAIN6 and VASCULAR-RELATED NAC-DOMAIN7 effectively induce transdifferentiation into xylem vessel elements under control of an induction system. Plant Physiol..

[13] Zhou J., Zhong R., Ye Z.H. (2014). Arabidopsis NAC domain proteins, VND1 to VND5, are transcriptional regulators of secondary wall biosynthesis in vessels. PLoS ONE.

[14] Nakano Y., Yamaguchi M., Endo H., Rejab N.A., Ohtani M. (2015). NAC-MYB-based transcriptional regulation of secondary cell wall biosynthesis in land plants. Front. Plant Sci..

[15] Ohtani M., Demura T. (2019). The quest for transcriptional hubs of lignin biosynthesis: Beyond the NAC-MYB-gene regulatory network model. Curr. Opin. Biotechnol..

[16] Yamaguchi M., Mitsuda N., Ohtani M., Ohme-Takagi M., Demura T. (2011). VASCULAR-RELATED NAC-DOMAIN7 directly regulates the expression of broad range of genes for xylem vessel formation. Plant J..

[17] Zhong R., Lee C., Ye Z.H. (2010). Global analysis of direct targets of secondary wall NAC master switches in Arabidopsis. Mol. Plant.

[18] Kim W.C., Ko J.H., Han K.H. (2012). Identification of a *cis*-acting regulatory motif recognized by MYB46, a master regulator of secondary wall biosynthesis. Plant Mol. Biol..

[19] Ko J.H., Jeon H.W., Kim W.C., Han K.H. (2014). The MYB46/MYB83-mediated transcriptional regulatory programme is a gatekeeper of secondary wall biosynthesis. Ann. Bot..

[20] Zhong R., Richardson E.A., Ye Z.H. (2007). The MYB46 transcription factor is a direct target of SND1 and regulates secondary cell wall biosynthesis in *Arabidopsis*. Plant Cell.

[21] Zhong R., Ye Z.H. (2012). MYB46 and MYB83 bind to the SMRE sites and directly activate a suit of transcription factors and secondary wall biosynthetic genes. Plant Cell Physiol..

[22] Nakano Y., Nishikubo N., Goué N., Ohtani M., Yamaguchi M., Katayama Y., Demura T. (2010). MYB transcription factors orchestrating the developmental program of xylem vessels in Arabidopsis roots. Plant Biotechnol..

[23] Xie M., Zhang J., Tschaplinski T.J., Tuskan G.A., Chen J.G., Muchero W. (2018). Regulation of lignin biosynthesis and its role in growth-defense tradeoffs. Front. Plant Sci..

[24] Zhou J., Lee C., Zhong R., Ye Z.H. (2009). MYB58 and MYB63 are transcriptional activators of the lignin biosynthetic pathway during secondary cell wall formation in *Arabidopsis*. Plant Cell.

[25] Xu B., Ohtani M., Yamaguchi M., Toyooka K., Wakazaki M., Sato M., Kubo M., Nakano Y., Sano R., Hiwatashi Y. (2014). Contribution of NAC transcription factors of plant adaptation to land. Science.

[26] Akiyoshi N., Nakano Y., Sano R., Kunigita Y., Ohtani M., Demura T. (2019). Involvement of VNS NAC-domain transcription factors in tracheid formation in *Pinus taeda*. Tree Physiol..

[27] Ohtani M., Nishikubo N., Xu B., Yamaguchi M., Mitsuda N., Goué N., Shi F., Ohme-Takagi M., Demura T. (2011). A NAC domain protein family contributing to the regulation of wood formation in poplar. Plant J..

[28] Zhong R., Lee C., Ye Z.H. (2010). Functional characterization of poplar wood associated NAC domain transcription factors. Plant Physiol..

[29] Yoshida K., Sakamoto S., Kawai T., Kobayashi Y., Sato K., Ichinose Y., Yaoi K., Akiyoshi-Endo M., Sato H., Takamizo T. (2013). Engineering the *Oryza sativa* cell wall with rice NAC transcription factors regulating secondary wall formation. Front. Plant Sci..

[30] Ohashi-Ito K., Iwamoto K., Fukuda H. (2018). LOB DOMAIN-CONTAINING PROTEIN 15 positively regulates expression of VND7, a master regulator of tracheary elements. Plant Cell Physiol..

[31] Soyano T., Thitamadee S., Machida Y., Chua N.H. (2008). ASYMMETRIC LEAVES2-LIKE19/LATERAL ORGAN BOUNDARIES DOMAIN30 and ASL20/LBD18 regulate tracheary element differentiation in *Arabidopsis*. Plant Cell.

[32] Yamaguchi M., Ohtani M., Mitsuda N., Kubo M., Ohme-Takagi M., Fukuda H., Demura T. (2010). VND-INTERACTING2, a NAC domain transcription factor, negatively regulates xylem vessel formation in *Arabidopsis*. Plant Cell.

[33] Kawabe H., Ohtani M., Kurata T., Sakamoto T., Demura T. (2018). Protein S-nitrosylation regulates xylem vessel cell differentiation in Arabidopsis. Plant Cell Physiol..

[34] Ohtani M., Kawabe H., Demura T. (2018). Evidence that thiol-based redox state is critical for xylem vessel cell differentiation. Plant Signal. Behav..

[35] Aoyama T., Chua N.H. (1997). A glucocorticoid-mediated transcriptional induction system in transgenic plants. Plant J..

[36] Lloyd A.M., Schena M., Walbot V., Davis R.W. (1994). Epidermal cell fate determination in Arabidopsis: Patterns defined by a steroid-inducible regulator. Science.

[37] Hasegawa J., Sakamoto Y., Nakagami S., Aida M., Sawa S., Matsunaga S. (2016). Three-dimensional imaging of plant organs using a simple and rapid transparency technique. Plant Cell Physiol..

[38] Higaki T., Kutsuna N., Akita K., Sato M., Sawaki F., Kobayashi M., Nagata N., Toyooka K., Hasezawa S. (2015). Semi-automatic organelle detection on transmission electron microscopic images. Sci. Rep..

[39] McCarthy R.L., Zhong R., Ye Z.H. (2009). MYB83 is a direct target of SND1 and acts redundantly with MYB46 in the regulation of secondary cell wall biosynthesis in Arabidopsis. Plant Cell Physiol..

[40] Bollhöner B., Zhang B., Stael S., Denancé N., Overmyer K., Goffner D., Van Breusegem F., Tuominen H. (2013). Post mortem function of AtMC9 in xylem vessel elements. New Phytol..

[41] Funk V., Kositsup B., Zhao C., Beers E.P. (2002). The Arabidopsis xylem peptidase XCP1 is a tracheary element vacuolar protein that may be a papain ortholog. Plant Physiol..

[42] Ko J.H., Kim W.C., Han K.H. (2009). Ectopic expression of MYB46 identifies transcriptional regulatory genes involved in secondary wall biosynthesis in Arabidopsis. Plant J..

[43] Brown D.M., Zeef L.A.H., Ellis J., Goodacre R., Turner S.R. (2005). Identification of novel genes in Arabidopsis involved in secondary cell wall formation using expression profiling and reverse genetics. Plant Cell.

[44] Peña M.J., Zhong R., Zhou G.K., Richardson E.A., O’Neil M.A., Darvill A.G., York W.S., Ye Z.H. (2007). Arabidopsis irregular xylem8 and irregular xylem9: Implications for the complexity of glucuronoxylan biosynthesis. Plant Cell.

[45] Persson S., Wei H., Milne J., Page G.P., Somerville C.R. (2005). Identification of genes required for cellulose synthesis by regression analysis of public microarray data sets. Proc. Natl. Acad. Sci. USA.

[46] Raes J., Rohde A., Christensen J.H., Van de Peer Y., Boerjan W. (2003). Genome-wide characterization of the lignification toolbox in Arabidopsis. Plant Physiol..

[47] Hussey S.G., Mizrachi E., Creux N.M., Myburg A.A. (2013). Navigating the transcriptional roadmap regulating plant secondary cell wall deposition. Front. Plant Sci..

[48] Takenaka Y., Watanabe Y., Schuetz M., Unda F., Hill J.L., Phookaew P., Yoneda A., Mansfield S.D., Samuels L., Ohtani M. (2018). Patterned deposition of xylan and lignin is independent from that of the secondary wall cellulose of Arabidopsis xylem vessels. Plant Cell.

[49] Porra R.J., Thompson W.A., Kriedemann P.E. (1989). Determination of accurate extinction coefficients and simultaneous equations for assaying chlorophylls *a* and *b* extracted with four different solvents: Verification of the concentration of chlorophyll standards by atomic absorption spectroscopy. Biochim. Biophys. Acta.

[50] Thévenaz P., Unser M. (2007). User-friendly semiautomated assembly of accurate image mosaics in microscopy. Microsc. Res. Tech..

[51] Chen Y.Q., Nixon M.S., Thomas D.W. (1995). Statistical geometrical features for texture classification. Pattern Recognit..

[52] Saeed A.I., Sharov V., White J., Li J., Liang W., Bhagabati N., Braisted J., Klapa M., Currier T., Thiagarajan M. (2003). TM4: A free, open-source system for microarray data management and analysis. Biotechniques.

